# Sex‐Related Differences in Histone Acetylation and Tumor Development in a 4‐Nitroquinoline 1‐Oxide and Ethanol‐Induced Oral Squamous Cell Carcinoma Mouse Model

**DOI:** 10.1111/jop.70062

**Published:** 2025-09-10

**Authors:** Wender Rodrigues Nazário, Anaíra Ribeiro Guedes Fonseca Costa, Débora de Oliveira Santos, Nayara Rúbio Diniz Del Nero, Tamiris Sabrina Rodrigues, Luiza Diniz Ferreira Borges, Carlos Ueira‐Vieira, Sérgio Vitorino Cardoso, Adriano Mota Loyola, Paulo Rogério de Faria

**Affiliations:** ^1^ Department of Oral and Maxillofacial Pathology Federal University of Uberlândia Uberlândia Minas Gerais Brazil; ^2^ Applied Immunology and Parasitology Postgraduate Program, Biomedical Sciences Institute Federal University of Uberlândia Uberlândia Minas Gerais Brazil; ^3^ Institute of Biotechnology Federal University of Uberlândia Patos de Minas Minas Gerais Brazil; ^4^ Institute of Biotechnology Federal University of Uberlândia Uberlândia Minas Gerais Brazil

**Keywords:** histone acetylation, mice, oral carcinogenesis, oral epithelial dysplasia, oral squamous cell carcinoma, sex differences

## Abstract

**Background:**

Oral squamous cell carcinoma (OSCC) is one of the most frequent head and neck cancers. The 4‐nitroquinoline 1‐oxide (4NQO) mouse model of oral carcinogenesis is a well‐established model to investigate the mechanism behind OSCC development, including epigenetic alterations. Studies have shown that histone acetylation is a key regulator of gene expression and may play a role in such a tumor. This study investigates the acetylation of H3K9, H3K14, and H3K27 and the *KAT2A* gene expression in a sex‐based approach in a 4NQO/ethanol (EtOH)‐induced OSCC mouse model.

**Methods:**

A total of 120 C57Bl/6J mice (60 males and 60 females) were divided into four groups (*n* = 15). In the first 10 weeks, they were treated with 5 mg/mL propylene glycol (PPG) or 100 μg/mL 4NQO in drinking water, followed by either sterilized water or 8% EtOH for 15 weeks. After euthanasia, tongues were analyzed histopathologically. Immunohistochemistry and qPCR were also employed to study the histones and gene expression.

**Results:**

Female mice showed increased H3K9ac and H3K14ac expression from normal mucosa to dysplasia, followed by decreased expression in OSCC. H3K9ac and H3K14ac expression in males was lower in the 4NQO/EtOH group. H3K27ac was higher in dysplastic lesions compared to OSCC, particularly in females. Comparatively, females had higher H3K9ac and H3K14ac expression in the 4NQO/EtOH group than males. *KAT2A* expression was lower in females treated with PPG/EtOH and 4NQO/H_2_O than in males.

**Conclusion:**

Our results indicate that H3 acetylation and *KAT2A* gene expression may play a key role in oral carcinogenesis on a sex‐related basis.

## 1 Introduction

Oral squamous cell carcinoma (OSCC) is one of the most common types of head and neck cancers, representing a significant public health issue due to its high mortality and morbidity [1]. Oral carcinogenesis is a multifactorial process influenced by genetic and epigenetic factors, as well as exposure to environmental carcinogens such as alcohol and tobacco [2, 3]. Recent studies have demonstrated that epigenetic alterations, including histone modifications, play a crucial role in tumor development and progression by influencing the expression of oncogenes and tumor suppressor genes [4, 5].

Histone acetylation is one of the major epigenetic modifications regulated by histone acetyltransferases (HATs) and histone deacetylases (HDACs) [6]. These modifications directly affect chromatin structure and gene transcription [6, 7]. In OSCC, acetylation of the histones H3K9, H3K14, and H3K27 is associated with transcriptional activation and gene silencing, directly impacting tumor development and progression [8, 9]. Experimental mouse models of oral carcinogenesis have been widely used to investigate the genetic and epigenetic mechanisms in OSCC development and progression [2, 10]. The 4‐nitroquinoline 1‐oxide (4NQO)‐induced oral carcinogenesis is a well‐established mouse model since it reproduces the molecular and histopathological characteristics of human OSCC, enabling the analysis of carcinogen effects such as ethanol (EtOH) [10]. Previous studies have demonstrated that chronic EtOH exposure contributes to epigenetic alterations, leading to increased OSCC incidence and tumor progression [3, 11]. In addition to epigenetic alterations, sex‐related differences have been investigated in oral carcinogenesis. Evidence suggests that sex hormones can modulate HAT and HDAC activity, influencing the epigenetic regulation of tumor development‐associated genes [12, 13]. The differential expression of the *KAT2A* gene, an important HAT involved in cell cycle regulation and genome stability, may play a fundamental role in OSCC susceptibility in a sex‐dependent manner [14].

The present study aimed to investigate the role of epigenetic modifications in the 4NQO/EtOH‐induced OSCC mouse model, focusing on the acetylated histones H3K9, H3K14, and H3K27 evaluated through immunohistochemistry and KAT2A gene expression, assessed via gene expression analysis. Additionally, sex‐related differences in these epigenetic markers were analyzed to better understand their effect on tumor development by comparing male and female mice exposed to the same carcinogens.

## 2 Materials and Methods

### 2.1 Study Design and Ethical Issues

Our experiment consisted of 120 specific‐pathogen‐free C57Bl/6J mice provided and maintained by the Institutional Rodents Animal Facilities Complex. This experimental study divided the animals into 60 male and 60 female mice, aged 6–8 weeks and weighing 25–30 g. Animals were kept in 32 × 20 × 21 cm micro‐isolator cages at 22°C ± 2°C, light–dark cycles of 12 h, with free access to sterilized water and food. At the beginning of the experiment, five mice were housed per cage with environmental enrichment. However, animal density per cage was reduced to at least two animals when aggressive behaviors were observed. All procedures were approved by the Ethical Commission on Animal Use (CEUA, Protocol Number 20/21).

### 2.2 Experimental Protocol

Epithelial dysplasia (ED) and OSCC were induced using two animal models: (1) the 4NQO (N8141, Sigma‐Aldrich, USA) model and (2) the Meadows–Cook chronic alcoholism model with 8% EtOH (95% Pa Acs, Êxodo Científica, Brazil), as previously described [15]. Treatments were administered *ad libitum* in a single bottle. For the first 10 weeks, animals received either 5 mg/mL propylene glycol (PPG, Synth, Brazil) or 100 μg/mL 4NQO diluted in PPG. Subsequently, they were treated with either sterilized water (H_2_O) or 8% EtOH (v/v) for 15 weeks. One hundred twenty mice (60 male, 60 female) were divided into four groups (*n* = 15): PPG/H_2_O, PPG/EtOH, 4NQO/H_2_O, and 4NQO/EtOH. The PPG/EtOH and 4NQO/EtOH groups underwent a 1‐week acclimatization with EtOH concentration increasing by 4% every 3 days until they achieved an 8%‐final concentration.

Animals were clinically evaluated over 25 weeks. Humane endpoints were applied weekly (Weeks 1–10) and daily (Weeks 11–25) when mice presented piloerection, ≤ 20% weight loss, inability to ambulate, labored respiration, dehydration, hunched posture, ocular/nasal discharge, and failure to access food/water. Euthanasia was performed via intraperitoneal injection of ketamine (300 mg/kg) and xylazine (30 mg/kg), followed by cervical dislocation. Tongues were collected and macroscopically inspected for lesions. Without lesions, the tongues were longitudinally sectioned in the midline. On the contrary, lesions‐containing tongues were sectioned longitudinally so that the sectioning plan passed through the largest lesion. One fragment was fixed in 7% paraformaldehyde for 24 h, processed, and embedded in paraffin for histology and immunohistochemistry. The other was snap‐frozen at −180°C for gene expression analysis.

### 2.3 Histopathological Analysis

A total of 120 scanned hematoxylin and eosin‐stained slides (×400 magnification, Aperio AT Turbo, Leica Biosystems, Nussloch, Germany) were analyzed by two experienced pathologists to identify areas of NM (normal mucosa), ED (low‐risk dysplasia [LRD] and high‐risk dysplasia [HRD]), and OSCC [16]. NM, ED, and OSCC were classified as previously reported [17].

### 2.4 Immunohistochemistry

The immunohistochemical assay was performed on 3‐μm‐thick sections using the Rabbit‐on‐Rodent HRP‐Polymer Detection System (Biocare Medical, Concord, CA). Peroxidase activity was blocked thrice with 10% hydrogen peroxide (v/v) for 10 min. Antigen retrieval was performed using Citrate Buffer (pH 6.0) in a pressure cooker at 100°C–110°C for 10 min. Slides were washed in PBS (pH 7.4), incubated with Rodent Block M (Biocare Medical) for 30 min at room temperature to block endogenous mouse IgG and nonspecific background, and then with primary antibodies in a humidity chamber at 4°C overnight. Antibodies used: H3K9ac (ab32129, Abcam; 1:1000), H3K14ac (ab52946, Abcam; 1:1000), and H3K27ac (ab177178, Abcam; 1:1000). Signal amplification and staining followed the Rabbit‐on‐Rodent HRP‐Polymer protocol. Sections were counterstained with Harris hematoxylin for 40 s. Positive controls were manufacturer‐recommended or literature‐supported. Negative controls omitted the primary antibody.

### 2.5 Immunohistochemical Analyses

Photomicrographs with a resolution of 2592 × 1944 pixels were captured using a 5.1 MP C‐Mount digital camera (Model: TA‐0120‐B) mounted on a Nikon E200 MVR microscope at ×400 magnification. For each sample, up to 10 areas of NM were photographed from PPG/H_2_O and PPG/EtOH mice, while 10 areas of LRD, HRD, and OSCC were sequentially captured from 4NQO/H_2_O and 4NQO/EtOH mice. The percentage of nuclear area with positive staining for each antibody was quantified using ImageJ software (version 1.54 m), following the methodology previously reported [18]. The average positive area was calculated based on all photomicrographs obtained for each lesion or control animal.

### 2.6 RNA Extraction, cDNA Synthesis, and RT‐qPCR Assays

Total RNA from tongue samples was extracted using TRI Reagent (AM9738, Thermo Fisher Scientific) and treated with 10U of RNase‐Free DNase I (Z3585, Promega Corporation). RNA concentration and quality were measured at 260 nm (ND‐1000 Spectrophotometer). From 1 μg of DNase‐treated RNA, cDNA was synthesized using Oligo (*dT*)15 (Exxtend, Brazil) and M‐MLV Reverse Transcriptase (M1701, Promega Corporation). Transcript levels of *KAT2A* were analyzed in triplicate by qPCR (QuantiNova SYBR Green PCR Kit, 208 052, Qiagen) using 5 pmol of specific primers. Cycling conditions: 50°C for 2 min, 95°C for 10 min, 40 cycles of 95°C for 15 s, 60°C for 30 s, 72°C for 30 s, followed by 95°C for 1 min and 55°C for 1 min. GAPDH was used for normalization. Product specificity was confirmed by melting curve analysis. Relative expression was calculated using the 2^−ΔΔC^_*t*_ method. Primer sequences and melting temperatures are listed in the Table S1.

### 2.7 Statistical Analyses

All analyses were performed in GraphPad Prism software version 8.2 (GraphPad, San Diego, California, USA) and R Statistical Software (version 4.4.2, R Core Team 2021) with an alpha value set at 5%. In the histopathological analysis, differences in the proportion of ED‐ and OSCC‐bearing animals according to sex and treatment groups were verified with Barnard's unconditional test. The distribution of ED, including LRD and HRD, and OSCC lesions between sex and treatment groups was analyzed with the *χ* ^2^ test. In the immunohistochemical analysis, differences in the percentage of positive nuclear area among groups were analyzed using the Kruskal–Wallis test with Dunn's post hoc test. Intragroup differences for each histological class were verified with Tukey's multiple comparisons test. The Mann–Whitney test was applied to analyze *KAT2A* expression.

## 3 Results

### 3.1 Histopathological Analysis

Table 1 describes the number of animals exhibiting the most severe lesion according to treatment groups. Except for a single female treated with 4NQO/H_2_O, who had no alterations in the tongue epithelium, all animals from both the 4NQO/H_2_O and 4NQO/EtOH groups developed either ED or OSCC. A lower number of OSCC‐bearing samples were observed in females treated with 4NQO/EtOH compared to males, while the latter had a lower number of animals with ED only. A similar pattern was observed in the 4NQO/H_2_O group, but the differences were insignificant. Furthermore, the females from the 4NQO/EtOH group had a higher and lower number of ED (LRD + HRD) and OSCC, respectively, than males when the total number of epithelial lesions observed per group was analyzed (Table 2).

**TABLE 1:** The number of animals exhibiting the most severe lesion per treatment group.

Histopathological class	Experimental groups, *n* (%)					*p*
	4NQO/H_2_O		*p*	4NQO/EtOH		
	M	F		M	F	
No alterations	0	1		0	0	
Low grade dysplasia	0	1	0.534	0	1	0.619
High grade dysplasia	1	3		1	5	
All epithelial dysplasia	1	4	0.092	1	6	0.026*
Oral squamous cell carcinoma	13	10		13	9	
Total	14	15		14	15	

*Note*: One‐sided Barnard's unconditional test, *p* < 0.05.

Abbreviations: 4NQO, 4‐nitroquinoline‐1‐oxide; EtOH, ethanol; F, female mice; H_2_O, water; M, male mice; *n*, number of animals.

* Statistically significant.

**TABLE 2:** The number of epithelial lesions observed per treatment group.

Histopathological lesions	Experimental groups, *n* (%)					*p*
	4NQO/H_2_O		*p*	4NQO/EtOH		
	M	F		M	F	
Low grade dysplasia	28	37	0.8869	24	52	0.9452
High grade dysplasia	20	25		18	38	
All epithelial dysplasia	48	52	0.2099	42	90	0.0005*
Oral squamous cell carcinoma	25	17		30	20	
Total	73	69		72	110	

*Note: χ* ^2^, *p* > 0.05.

Abbreviations: 4NQO, 4‐nitroquinoline‐1‐oxide; EtOH, ethanol; F, female mice; H_2_O, water; M, male mice; *n*, number of animals.

* Statistically significant.

### 3.2 Immunohistochemical Expression

#### 3.2.1 H3K9ac

**FIGURE 1:**
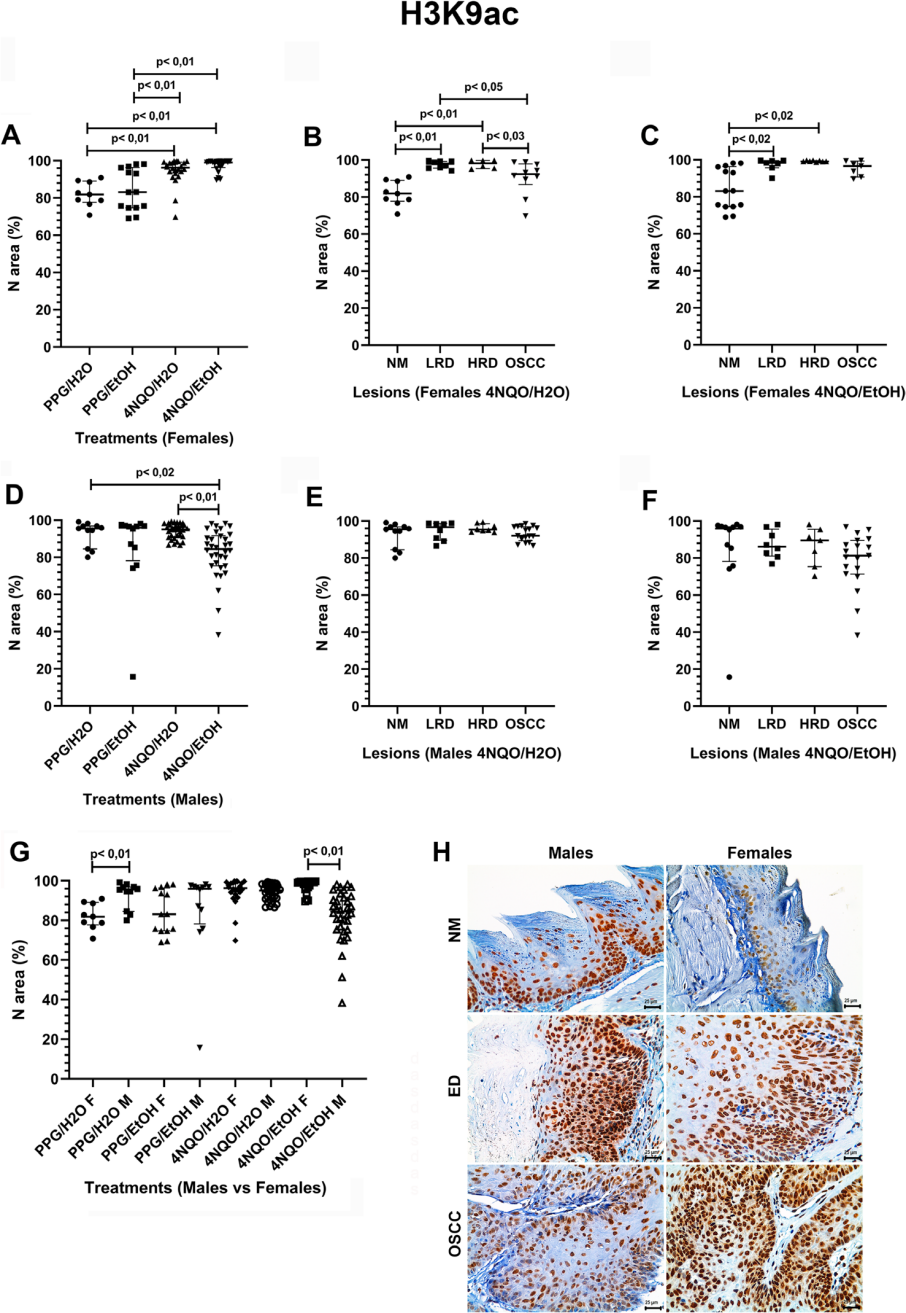
Comparative analysis of H3K9ac expression in nuclear area percentage (N area %) across experimental groups and histologically classified lesions. (A) Nuclear expression of H3K9ac in females across all treatment groups. (B) Nuclear expression of H3K9ac in females from the 4NQO/H_2_O group, stratified by lesion type. (C) Nuclear expression of H3K9ac in females from the 4NQO/EtOH group, stratified by lesion type. (D) Nuclear expression of H3K9ac in males across all treatment groups. (E) Nuclear expression of H3K9ac in males from the 4NQO/H_2_O group, stratified by lesion type. (F) Nuclear expression of H3K9ac in males from the 4NQO/EtOH group, stratified by lesion type. (G) Comparative analysis of nuclear H3K9ac expression between males (M) and females (F) under the same treatment. (H) Photomicrographs showing H3K9ac expression in tongue tissue samples from male and female mice, including NM, ED, and OSCC (original magnification ×400). Statistical significance was determined using the Kruskal–Wallis test with Dunn's post hoc test (*p* < 0.05). 4NQO, 4‐nitroquinoline‐1‐oxide; ED, epithelial dysplasia; EtOH, ethanol; H_2_O, water; HRD, high‐risk dysplasia; LRD, low‐risk dysplasia; N area %; nuclear area percentage; NM, normal mucosa; OSCC, oral squamous cell carcinoma; PPG, propylene glycol.

Females from the 4NQO/H_2_O and 4NQO/EtOH groups showed a significantly higher H3K9ac expression than the PPG/H_2_O and PPG/EtOH ones (Figure 1A). In the 4NQO/H_2_O group, the H3K9ac expression increased from the NM to ED (LRD and HRD) lesions but reduced from the ED to OSCC ones (Figure 1B). In the 4NQO/EtOH group, the ED also had higher H3K9ac immunostaining than the NM, although differences were insignificant compared to the OSCC lesions (Figure 1C).

Males from the 4NQO/EtOH group had a lower H3K9ac than the PPG/H_2_O and 4NQO/H_2_O groups (Figure 1D). In the 4NQO/H_2_O group, OSCC‐bearing males had a higher H3K9ac expression than the other lesions, though it did not achieve significance (Figure 1E). In the 4NQO/EtOH group, the NM had a higher H3K9ac than the LRD, HRD, and OSCC lesions, but without significance (Figure 1F). Comparatively, females from the PPG/H_2_O group had a lower H3K9ac expression than males, while the inverse was observed for the females from the 4NQO/EtOH group (Figure 1G).

Figure 1H illustrates the H3K9ac expression in the NM, ED, and OSCC in male and female mice. In NM, H3K9ac immunoreactivity ranged from weak to moderate within keratinocyte nuclei, with rare cytoplasmic staining. In ED and OSCC, diffuse nuclear staining varied from weak to strong. ED frequently presented negative cells in basal/parabasal layers, while OSCC lesions exhibited heterogeneous nuclear reactivity.

#### 3.2.2 H3K14ac

**FIGURE 2:**
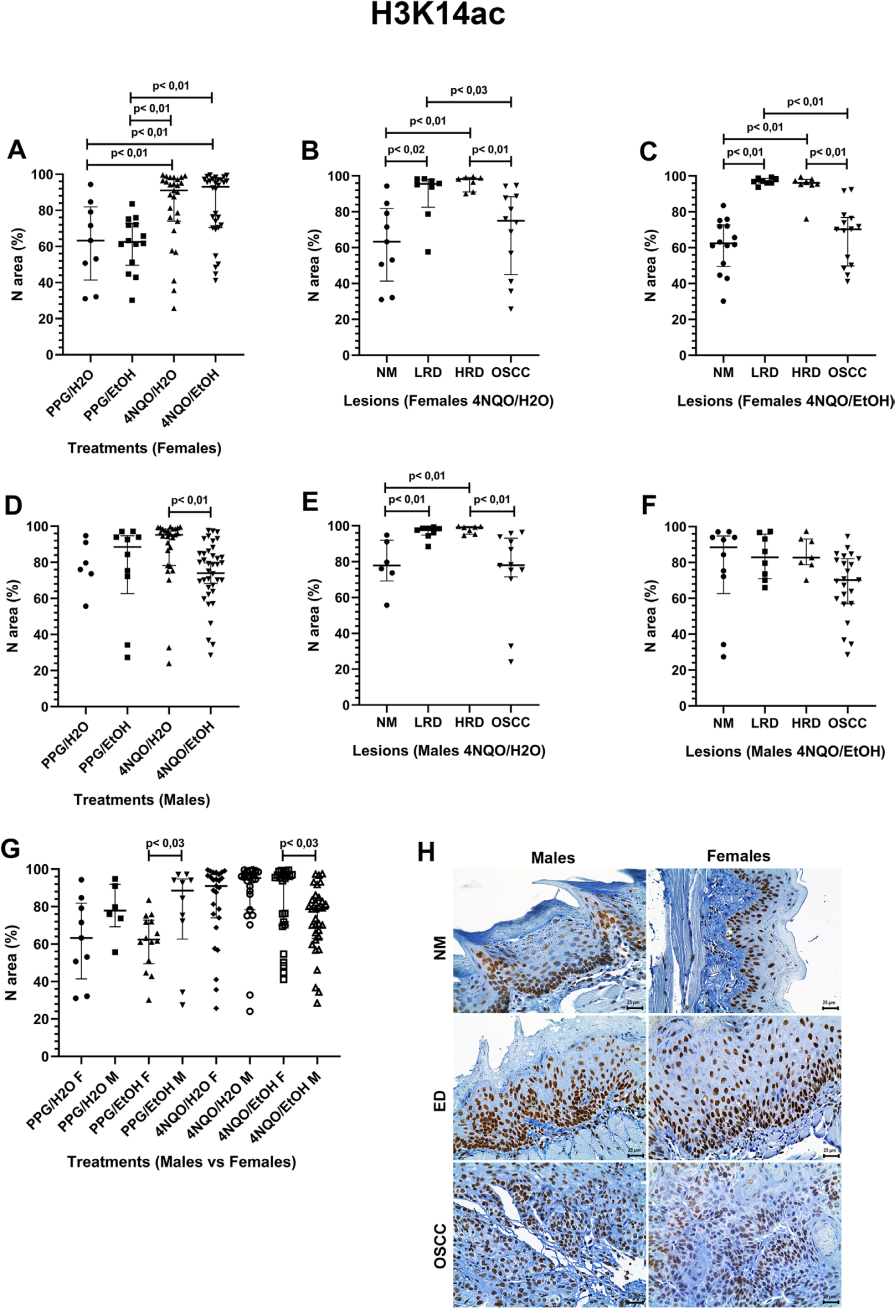
Comparative analysis of H3K14ac expression in nuclear area percentage (N area %) across experimental groups and histologically classified lesions. (A) Nuclear expression of H3K14ac in females across all treatment groups. (B) Nuclear expression of H3K14ac in females from the 4NQO/H_2_O group, stratified by lesion type. (C) Nuclear expression of H3K14ac in females from the 4NQO/EtOH group, stratified by lesion type. (D) Nuclear expression of H3K14ac in males across all treatment groups. (E) Nuclear expression of H3K14ac in males from the 4NQO/H_2_O group, stratified by lesion type. (F) Nuclear expression of H3K14ac in males from the 4NQO/EtOH group, stratified by lesion type. (G) Comparative analysis of nuclear H3K14ac expression between males (M) and females (F) under the same treatment. (H) Photomicrographs showing H3K14ac expression in tongue tissue samples from male and female mice, including NM, ED, and OSCC (original magnification ×400). Statistical significance was determined using the Kruskal‐Wallis test with Dunn's post hoc test (*p* < 0.05). 4NQO, 4‐nitroquinoline‐1‐oxide; ED, epithelial dysplasia; EtOH, ethanol; H_2_O, water; HRD, high‐risk dysplasia; LRD, low‐risk dysplasia; N area %; nuclear area percentage; NM, normal mucosa; OSCC, oral squamous cell carcinoma; PPG, propylene glycol.

Females from the 4NQO/H_2_O and 4NQO/EtOH groups had a higher H3K14ac expression than those from the PPG/H_2_O and PPG/EtOH groups (Figure 2A). In the 4NQO/H_2_O group, the H3K14ac expression increased from the NM to the ED but decreased in the OSCC lesions (Figure 2B). Similar patterns were observed in the 4NQO/EtOH group (Figure 2C).

Males from the 4NQO/H_2_O group had a higher H3K14ac expression than those from the 4NQO/EtOH group (Figure 2D). In addition, the H3K14ac expression increased from the NM to the ED lesion but decreased in the OSCC ones (Figure 2E). A different expression pattern was observed in the 4NQO/EtOH group, though a reduction in H3K14ac expression was also noted in OSCC lesions (Figure 2F). Comparatively, females from the 4NQO/EtOH group had a higher H3K14ac expression than males of the same group. On the contrary, in males from the PPG/EtOH group, the expression of such a histone modification was higher than in females of the same group (Figure 2G).

Figure 2H shows H3K14ac expression across all groups. Strong nuclear staining was observed in NM in basal and suprabasal layers. In contrast, ED and OSCC samples exhibited heterogeneous nuclear expression, with reduced or absent staining areas.

#### 3.2.3 H3K27ac

**FIGURE 3:**
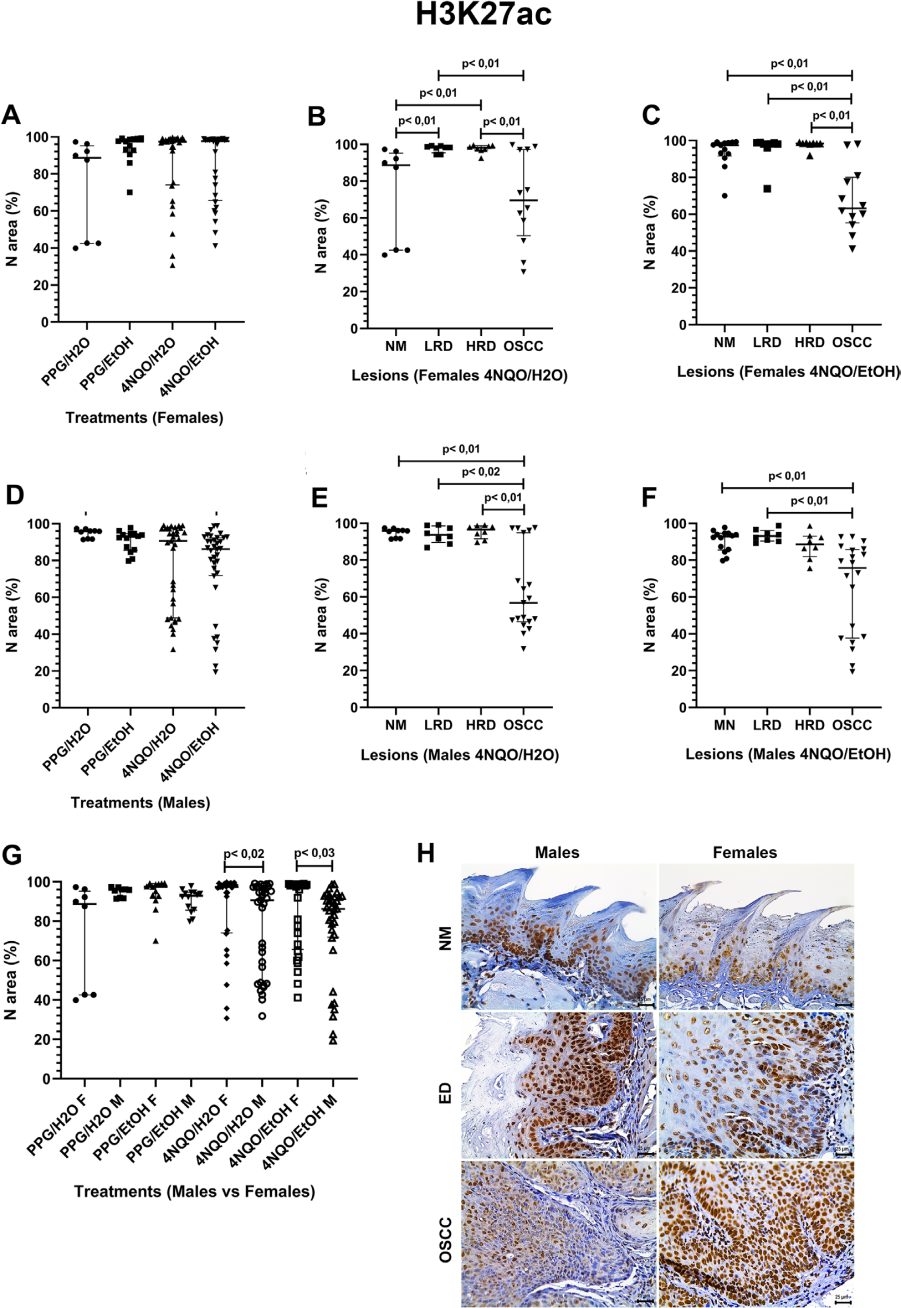
Comparative analysis of H3K27ac expression in N area % across experimental groups and histologically classified lesions. (A) Nuclear expression of H3K27ac in females across all treatment groups. (B) Nuclear expression of H3K27ac in females from the 4NQO/H2O group, stratified by lesion type. (C) Nuclear expression of H3K27ac in females from the 4NQO/EtOH group, stratified by lesion type. (D) Nuclear expression of H3K27ac in males across all treatment groups. (E) Nuclear expression of H3K27ac in males from the 4NQO/H_2_O group, stratified by lesion type. (F) Nuclear expression of H3K27ac in males from the 4NQO/EtOH group, stratified by lesion type. (G) Comparative analysis of nuclear H3K27ac expression between males (M) and females (F) under the same treatment. (H) Photomicrographs showing H3K27ac expression in tongue tissue samples from male and female mice, including NM, ED, and OSCC (original magnification ×400). Statistical significance was determined using the Kruskal‐Wallis test with Dunn's post hoc test (*p* < 0.05). 4NQO, 4‐nitroquinoline‐1‐oxide; ED, epithelial dysplasia; EtOH, ethanol; H_2_O, water; HRD, high‐risk dysplasia; LRD, low‐risk dysplasia; N area %; nuclear area percentage; NM, normal mucosa; OSCC, oral squamous cell carcinoma; PPG, propylene glycol.

Females from the PPG/EtOH, 4NQO/H_2_O, and 4NQO/EtOH groups showed a slight increase in the H3K27ac expression compared to those from the PPG/H_2_O group, but without significance (Figure 3A). In the 4NQO/H_2_O group, the LRD and HRD lesions had a higher H3K27ac expression than the NM and the OSCC lesions (Figure 3B). In the 4NQO/EtOH group, H3K27ac expression in the NM, LRD, and HRD was similar but significantly higher than in the OSCC lesions (Figure 3C).

Males showed a similar H3K27ac expression level across the groups, except for the PPG/H_2_O group, which exhibited the highest expression, and the 4NQO/EtOH group, which showed the lowest expression (Figure 3D). In the 4NQO/H_2_O group, the NM, HRD, and LRD had a higher H3K27ac expression than the OSCC lesions (Figure 3E). Regarding the 4NQO/EtOH group, H3K27ac expression in the NM, LRD, and HRD was also similar but significantly higher than in the OSCC lesions (Figure 3F). Comparatively, females from the 4NQO/H_2_O and 4NQO/EtOH groups had a higher H3K27ac expression than males of the same groups (Figure 3G).

Figure 3H shows H3K27ac expression in NM, ED, and OSCC. In NM, immunoreactivity was weak and predominantly nuclear in keratinocytes, particularly in the filiform papillae. ED and OSCC exhibited variable nuclear staining, including negative areas.

### 3.3 Gene Expression

#### 3.3.1 KAT2A

**FIGURE 4:**
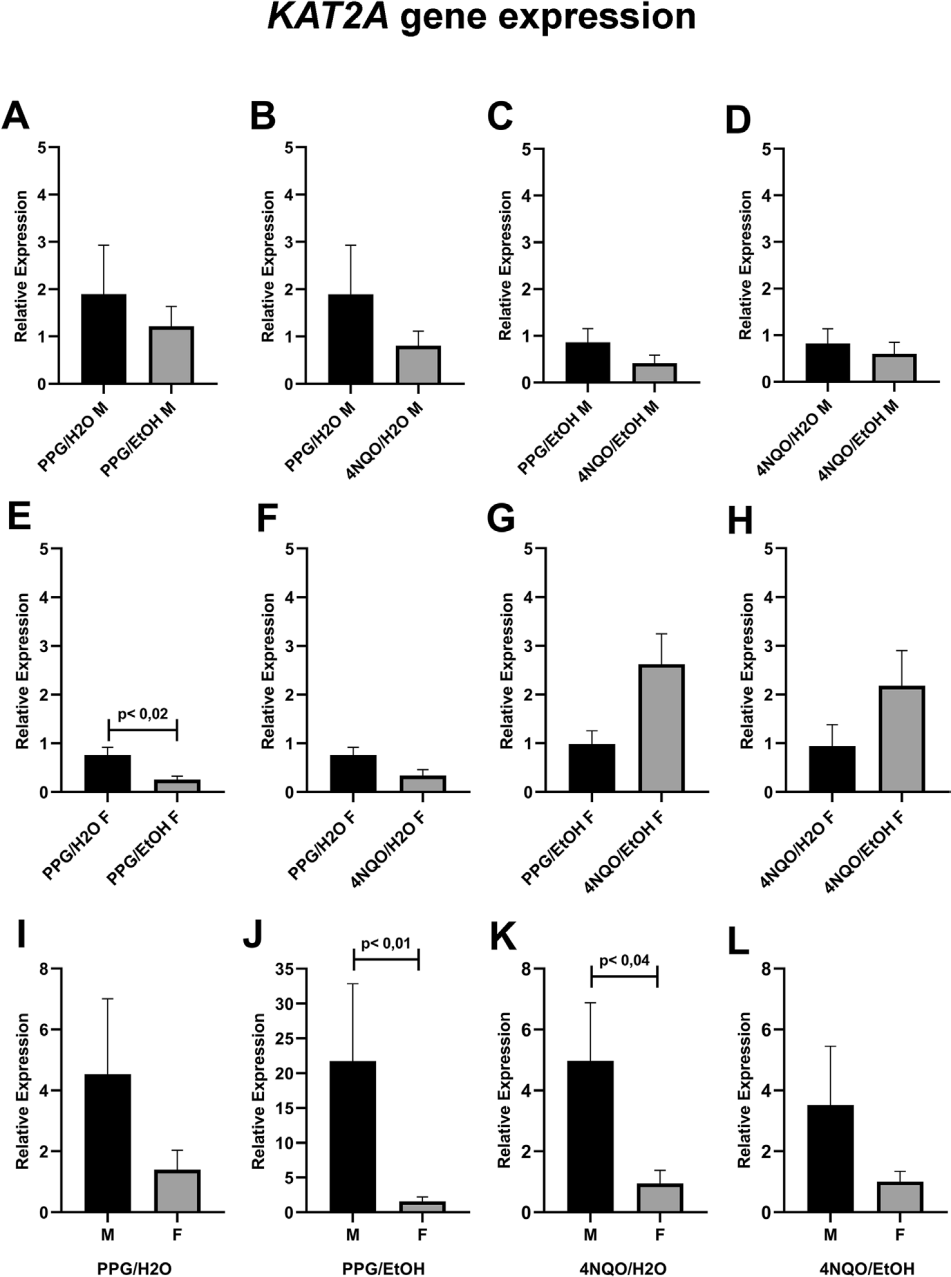
Comparative analysis of KAT2A relative expression across experimental groups. (A–D) Relative expression of KAT2A in males: (A) PPG/H_2_O versus PPG/EtOH, (B) PPG/H_2_O versus 4NQO/H_2_O, (C) PPG/EtOH versus 4NQO/EtOH, (D) 4NQO/H_2_O versus 4NQO/EtOH. (E–H) Relative expression of KAT2A in females: (E) PPG/H_2_O versus PPG/EtOH, (F) PPG/H_2_O versus 4NQO/H_2_O, (G) PPG/EtOH versus 4NQO/EtOH, (H) 4NQO/H_2_O versus 4NQO/EtOH. (I–L) Comparison of KAT2A expression between males and females: (I) PPG/H_2_O, (J) PPG/EtOH, (K) 4NQO/H_2_O, (L) 4NQO/EtOH. Statistical significance was determined using the Mann–Whitney test (*p* < 0.05). 4NQO, 4‐nitroquinoline‐1‐oxide; EtOH, ethanol; F, female; H_2_O, water; M, male; PPG, propylene glycol.

The relative gene expression of *KAT2A* decreased in males from the PPG/EtOH and 4NQO/H_2_O groups compared to the PPG/H_2_O one, though it did not achieve significance (Figure 4A–D). In females, *KAT2A* expression was reduced in the PPG/EtOH and the 4NQO/H_2_O groups compared to the PPG/H_2_O one, with significance only in the former (Figure 4E,F). Combining 4NQO with EtOH resulted in increased *KAT2A* expression compared to EtOH or 4NQO alone, although this increase did not reach statistical significance (Figure 4G,H). *KAT2A* expression was significantly lower in females from the PPG/EtOH and 4NQO/H_2_O groups compared with males in the same groups (Figure 4J,K). Likewise, females from the PPG/H_2_O and 4NQO/EtOH groups also showed reduced *KAT2A* expression compared with males, but this difference did not reach statistical significance (Figure 4I,L).

## 4 Discussion

Our study highlights alterations in H3K9ac, H3K14ac, H3K27ac, and *KAT2A* gene expression in the tongue lesions of male and female mice treated with the chemical initiator (4NQO) and promoter (EtOH) of oral carcinogenesis. The 4NQO model was chosen based on its efficacy in inducing oral carcinogenesis in rodents, the gold‐standard model to reproduce human OSCC development [10]. Similar approaches have been used to assess EtOH's impact on tumor development and epigenetic changes [2, 3].

Alcohol‐ and tobacco‐induced epigenetic modifications have been widely studied in human oral carcinomas [1, 19]. Ghantous et al. found that chronic exposure to these factors causes changes in histone modifications, including increased H3K9ac, H3K14ac, and H3K27ac expression levels [1]. These findings align with our observations of dynamic histone acetylation patterns in chemically induced lesions, suggesting that alcohol and tobacco are the major drivers of oral carcinogenesis through epigenetic alterations.

Histone acetylation, a key posttranslational modification, regulates chromatin processes like nucleosome organization and gene transcription [4, 20]. HATs and HDACs‐mediated acetylation of the histone lysine residues can influence cellular phenotypes [5, 6]. During carcinogenesis, these changes can activate oncogenes or silence tumor suppressor genes [7]. Moreover, sex‐related epigenetic differences also affect tumor development [12].

Urvalek et al. reported changes in H3K9ac, H3K14ac, and H3K27ac due to EtOH exposure, highlighting their role in alcohol‐associated carcinogenesis [2]. However, their study was limited to the treatment groups, while ours stratified lesions by histopathology, offering a more detailed evaluation of the epigenetic landscape. We found that dysplastic lesions showed increased H3K14ac and H3K27ac expression. At the same time, OSCCs exhibited reduced levels of the same histones, suggesting that the roles of histone acetylation may vary depending on the carcinogenesis stage. Sex‐related differences in histone modifications, not addressed by Urvalek et al., add a novel dimension to our findings [2].

Guo et al. showed that EtOH (8%) increased OSCC incidence in a 4NQO‐induced mouse model, but their study focused on lesion incidence and inflammatory pathways [3]. In contrast, our study examined histone modifications and sex‐related epigenetic differences. We also observed sex‐specific variations in lesion frequency, indicating epigenetic changes may influence tumor susceptibility. This finding highlights the importance of integrating molecular and epigenetic analyses to understand EtOH‐associated carcinogenesis.

In our investigation, the H3K9ac expression exhibited a heterogeneous pattern, with statistically significant differences between experimental groups and the types of lesions analyzed. Previous evidence showed that H3K9ac is associated with transcriptional activation in various malignancies, including an increased expression in OSCC [11]. However, reduced H3K9ac expression in OSCC lesions in our study corroborates previous reports that highlighted hypoacetylation in OSCC and its correlation with unfavorable prognosis [21]. These findings reinforce the hypothesis that low H3K9ac expression may be related to advanced tumor progression [21].

Regarding H3K14ac expression, our results agree with the Armenta‐Castro et al. study, indicating that H3K14 acetylation is linked with critical cellular process regulation, as observed in cervical cancer cells [8]. In our study, dysplasia lesions exhibited an increased H3K14ac expression, while OSCCs showed a significant reduction. This reduction suggests that enhanced H3K14 acetylation expression may be linked to higher oncogene transcriptional activities, followed by its reduction in OSCC and, thus, silencing of tumor suppressor genes [9, 22].

H3K27ac expression also displayed a heterogeneous pattern, with an increase in dysplasia and a decrease in OSCC. The literature describes H3K27ac as a super‐enhancer marker, often associated with transcriptional activation in oncogenes [23, 24]. However, the reduction observed in OSCCs suggests a dynamic role in transcriptional regulation during tumor progression, in agreement with our results. Previous studies suggest that the loss of H3K27ac in advanced cancer stages may be associated with epigenetic silencing of tumor suppressor genes [25, 26]. Additionally, the study by Liu et al. demonstrated that H3K27ac is involved in transcription, DNA repair, and replication in OSCC cells treated with metformin [27]. This finding emphasizes the importance of H3K27ac in tumor progression, particularly in cancer models with epigenetic alterations. The reduction of H3K27ac observed in OSCCs in the present study may be associated with the inactivation of tumor suppressor genes and reduced DNA repair, contributing to genomic instability.

*KAT2A* expression was notably lower in female lesions, suggesting a sex‐specific role in oral carcinogenesis. Sexual differences were observed in H3K9ac, H3K14ac, H3K27ac, and *KAT2A* expression, with females showing higher histone acetylation but lower *KAT2A* levels. These findings suggest sex hormones may modulate histone acetylation, influencing tumor development [12, 13, 28]. Lower OSCC incidence in females implies that low *KAT2A* expression may have a protective role. It is known that sex hormones can modulate HAT and HDAC activity through posttranslational modifications, influencing epigenetic regulation in cancer [29, 30]. Whether these mechanisms apply to oral carcinogenesis in mice remains to be investigated.

## 5 Conclusion

Our data highlight the dynamic nature of epigenetic modifications in the histones H3K9ac, H3K14ac, and H3K27 and *KAT2A* gene expression during a mouse model of oral carcinogenesis. The differential expression of these markers reinforces their potential as biomarkers, while the observed sexual differences underline the importance of considering biological heterogeneity in oral carcinogenesis. Although our findings in mouse models provide valuable insights, validation in human‐derived dysplastic and OSCC lesions is crucial to confirm the role of such histones and the *KAT2A* gene in developing such a tumor.

## Author Contributions

Conceptualization: Paulo Rogério de Faria and Adriano Mota Loyola. Methodology: Paulo Rogério de Faria, Adriano Mota Loyola, Anaíra Ribeiro Guedes Fonseca Costa, Débora de Oliveira Santos, Wender Rodrigues Nazário, Tamiris Sabrina Rodrigues, Luiza Diniz Ferreira Borges, and Carlos Ueira‐Vieira. Validation: Débora de Oliveira Santos, Tamiris Sabrina Rodrigues, Paulo Rogério de Faria, Sérgio Vitorino Cardoso, and Nayara Rúbio Diniz Del Nero. Formal analysis: Wender Rodrigues Nazário, Tamiris Sabrina Rodrigues, Anaíra Ribeiro Guedes Fonseca Costa, Paulo Rogério de Faria, and Sérgio Vitorino Cardoso. Investigation: Débora de Oliveira Santos, Anaíra Ribeiro Guedes Fonseca Costa, and Wender Rodrigues Nazário. Data curation: Wender Rodrigues Nazário. Writing – original draft: Wender Rodrigues Nazário, Anaíra Ribeiro Guedes Fonseca Costa, Débora de Oliveira Santos, and Paulo Rogério de Faria. Writing – review and editing: Anaíra Ribeiro Guedes Fonseca Costa, Adriano Mota Loyola, Sérgio Vitorino Cardoso, Paulo Rogério de Faria, and Nayara Rúbio Diniz Del Nero. Funding acquisition: Adriano Mota Loyola. Resources: Adriano Mota Loyola and Paulo Rogério de Faria. Supervision: Adriano Mota Loyola and Paulo Rogério de Faria. All authors approved the submitted version to be published.

## Ethics Statement

All procedures followed the institution's ethical standards where the studies were conducted. Ethical approval was obtained from the Ethical Commission in Animal Experimentation (CEUA‐UFU, number 020/21) on October 15, 2021.

## Conflicts of Interest

The authors declare no conflicts of interest.

## Supporting information


**Table S1:** Primer sequences and annealing temperatures in Celsius degrees.

## Data Availability

Data are available upon request.
