# Supplementary material for: Sex‐Related Differences in Histone Acetylation and Tumor Development in a 4‐Nitroquinoline 1‐Oxide and Ethanol‐Induced Oral Squamous Cell Carcinoma Mouse Model
Source: J Oral Pathol Med. 2025 Sep 10;54(10):1074–84. doi: 10.1111/jop.70062 (PMC12602132; doi:10.1111/jop.70062)
Supplement: Supplementary file 1 — Table S1: Primer sequences and annealing temperatures in Celsius degrees. [file JOP-54-1074-s001.docx]

**SUPPLEMENTAL MATERIAL**

**Supplementary Table 1:** Primer sequences and annealing temperatures in Celsius degrees.

| **Gene** | **AT (°C)** | **Forward** | **Reverse** |
| --- | --- | --- | --- |
| GAPDH | 62 | AGGTCGGTGTGAACGGATTTG | TGTAGACCATGTAGTTGAGGTCA |
| KAT2A | 60 | CGAGTTGTGCCGTAGCTGTGA | ACCATTCCCAAGAGCCGGTTA |
